# Efficacy and Safety of Haidebao Body Lotion in Patients With Mild Atopic Dermatitis: Protocol for a Multicenter, Double-Blind, Randomized, Placebo-Controlled Trial

**DOI:** 10.2196/71255

**Published:** 2025-06-10

**Authors:** Zhen Duan, Yuning Ding, Ruiping Wang

**Affiliations:** 1 Clinical Research Center, Shanghai Skin Diseases Hospital School of Medicine Tongji University Shanghai China; 2 School of Public Health Shanghai University of Traditional Chinese Medicine Shanghai China

**Keywords:** atopic dermatitis, emollient, randomized controlled trial, study protocol, double blind, double-blind

## Abstract

**Background:**

Atopic dermatitis (AD) is a chronic, relapsing skin condition that significantly impacts patients’ quality of life. In clinical practice, AD is commonly managed through the use of emollients and topical corticosteroids. Haidebao Body Lotion (HBL) incorporated with calcium-based antimicrobial peptide compounds (CAPCS) has demonstrated clinical benefits for patients with mild AD, but there is a lack of high-quality clinical trial evidence.

**Objective:**

In this study, we will implement a multicenter, double-blind, randomized, placebo-controlled trial to evaluate the efficacy and safety of HBL incorporated with CAPCS as an adjunctive therapy in ameliorating mild AD.

**Methods:**

This multicenter, double blind, randomized, placebo-controlled trial will recruit 200 eligible participants from 10 hospitals in China from October 2023 to October 2025. In this study, AD will be confirmed in accordance with Williams diagnostic criteria, and patients with mild AD, aged 18-55 years, who provide signed informed consent will be enrolled. However, patients who are pregnant, have serious underlying diseases, have communication barriers, and violate medication regulations will be excluded. The 200 patients will be randomly assigned (1:1) to a treatment group (HBL with CAPCS; n=100, 50%) and a control group (HBL without CAPCS, placebo; n=100, 50%), and each participant will receive 3 sessions of treatments per day for 4 weeks. The primary outcome is the proportion of patients who achieve at least 60% improvement in the Eczema Area and Severity Index (EASI) score from baseline to week 2. The secondary outcomes include the Numeric Rating Scale (NRS), EASI50, EASI60, and Dermatology Life Quality Index (DLQI) scores at weeks 2 and 4, as well as adherence and adverse events. The full analysis set (FAS) and the per protocol set (PPS) will be analyzed using SAS 9.3 software, and missing data will be processed using the multiple imputation method. In this study, *P*<.05 is considered statistically significant.

**Results:**

Participant recruitment began in January 2024. As of May 2025, we enrolled 180 patients, with 160 (88.9%) completing the 2-week follow-up. Data collection and management are still ongoing, and data analysis has not yet been performed.

**Conclusions:**

This study will evaluate the clinical efficacy and safety of HBL incorporated with CAPCS in the treatment of patients with mild AD. If treatment efficacy is proven, HBL incorporated with CAPCS could be clinically used as an adjunctive therapy in ameliorating mild AD.

**International Registered Report Identifier (IRRID):**

DERR1-10.2196/71255

## Introduction

Atopic dermatitis (AD) is a chronic, recurrent, inflammatory skin condition that affects approximately 11%-20% of children and 5%-8% of adults [1,2]. AD often begins in infancy, with approximately 50% of cases occurring before the age of 1 year [3]. AD usually exhibits a chronic course, occasionally protracts into adulthood, and is distinctly characterized by acute erythema featuring vague borders, accompanied by edema, blisters, and oozing blood [4]. In the chronic phase of AD, there is thickening of the skin, particularly in areas such as the cheeks, arms, and flexor surfaces of the trunk, which can result in sleep disturbances, severe life quality interference and daily activity deprivation, and the loss of psychological well-being [5,6]. AD is often associated with other allergic conditions, such as asthma and food allergies, and it may also increase the risk of nonallergic comorbidities, including various autoimmune or immune-mediated disorders, and psychiatric illnesses [7]. The persistent relapsing trajectory, elevated comorbidity rate, substantial economic burden, and involvement of the entire family in the treatment process have significantly reduced the quality of life of patients with AD and their family members [8,9].

The pathogenesis of AD is complex, involving genetic susceptibility, microbiome effects, environmental factors, and immune dysregulation [10]. Anti-inflammatory agents, phototherapy, wet-wrap therapy, systemic immunosuppressants, and short-term systemic steroid treatment are used to manage persistent or severe cases of AD [11]. However, use of the aforementioned local and systematic AD treatments often fails to provide long-term disease control due to safety concerns. Previous studies indicate that oral corticosteroids can ameliorate AD lesions, but the disease may flare up again if these medications are discontinued [12]. Moreover, topical steroids may cause side effects, involving skin thinning, lines, acne, cataracts, glaucoma, and delayed growth in children [13]. The use of antihistamines may lead to an increase in attention deficit hyperactivity disorder (ADHD) in children [14]. These side effects induced by local or systematic treatments restrict prolonged application of antihistamines in patients with AD.

The external application of moisturizing emollients is the basis for AD treatment, which can not only improve cuticle hydration and reduce inflammation [15] but also repair the damaged skin barrier, decrease the stimulation of external factors, and, thus, reduce the attack frequency and disease severity [16]. In patients with mild AD, continuous, frequent, and heavy use of emollients is recommended to maintain skin barrier function, even in the absence of lesions [17]. In recent years, the development of new active ingredients has led to the widespread clinical application of emollients to improve the skin barrier, with reparative, anti-inflammatory, and moisturizing effects, especially in patients with mild AD [18,19].

Calcium-based antimicrobial peptide compounds (CAPCS) consist of naturally occurring active calcium and a variety of antibacterial peptides (AMPs) derived from farmed marine shellfish. Previous studies indicate that active calcium can enhance capillary wall density, reduce capillary permeability and exudation, and safeguard the integrity of cell membranes. Simultaneously, active calcium can inhibit the release of inflammatory mediators and exhibit anti-inflammatory and anti-allergic properties. Previous studies indicate that calcium exists in the human body in the form of calcium ions. Calcium ions and their concentration gradients in the epidermis play a crucial role in regulating various skin functions, including keratinocyte differentiation, skin barrier formation, and permeability barrier homeostasis [20]. G protein–coupled receptors (GPCRs), which are widely expressed in mammals, are associated with skin pruritus through their signaling pathways. So, calcium ions can bind to G proteins, thereby inhibiting their activity and alleviating pruritus [21]. Moreover, the AMPs in CAPCS can alter bacterial cell membrane permeability, creating openings that allow calcium ions to rapidly enter the cells. This influx of calcium ions raises intracellular calcium levels, causing metabolic disruption and organelle leakage, ultimately leading to the death of pathogenic bacteria [22]. Additionally, AMPs can prevent bacterial infections, alleviate skin inflammatory responses, and thereby further improve patient symptoms [23]. Previous experimental studies indicate that CAPCS have therapeutic effects on AD, with similar efficacy as desonide cream (a weak corticosteroid) in controlling scratching and relieving itching in mice [24]. Haidebao Body Lotion (HBL) incorporated with CAPCS has also demonstrated clinical benefits for patients with mild AD, but there is a lack of high-quality clinical trial evidence. Therefore, this study aims to evaluate the treatment efficacy and safety of HBL incorporated with CAPCS in patients with mild AD.

## Methods

### Study Design

This study is a multicenter, double-blind, randomized, placebo-controlled clinical trial that will be conducted in 10 tertiary hospitals in China from October 2023 to October 2025. The 10 hospitals are the Shanghai Skin Diseases Hospital, the Third Hospital affiliated to Chongqing Medical University, the First Hospital of Hangzhou, the Third Hospital affiliated to Peking University, the First Hospital of Fujian Medical University, the Skin Diseases Hospital affiliated to South Medical University, the First Hospital of Wuhan, the First Hospital affiliated to Kunming Medical University, the Skin Diseases Hospital of Jiangxi, and the First Hospital affiliated to Anhui University of traditional Chinese medicine. In this study, patients with mild AD who provide signed informed consent will be randomly assigned (1:1) to a treatment group (HBL with CAPCS) and a control group (HBL without CAPCS, or placebo). This study is registered with the Chinese Clinical Trial Registry (ChiCTR2400087274).

### Sample Size

In this study, the sample size was calculated based on the proportion of patients achieving at least 60% improvement (defined as the effective rate) in the Eczema Area and Severity Index (EASI) score from baseline to week 2 (EASI_60_) in both the treatment group and the control group. A previous pilot study (data not published) indicated that the proportion of EASI_60_ achievement at week 2 was 50% in the CAPCS group and 20% in the placebo group. In addition, a previous open-label clinical trial involving 60 pediatric patients with AD demonstrated comparable efficacy outcomes, with CAPCS combination therapy showing a 42.86% response rate versus 7.14% for the control group at week 2, with statistically significant differences (*P*<.05) [25]. In this study, we used the following formula to calculate the sample size:




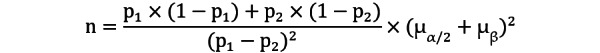




We set the proportion of EASI_60_ achievement at week 2 in the treatment group to p1=45%, the proportion of EASI_60_ achievement at week 2 in the control group to p2=20%, the type I error to α=0.05, the type II error to β=0.1. The sample size calculation indicated that at least 70 patients be recruited in each group. Considering a 20% dropout rate and a 10% multicenter effect, we plan to enroll 100 patients with mild AD in each group, for a total of 200 patients with mild AD.

### Participants

In this study, AD will be confirmed in accordance with Williams diagnostic criteria [26], and patients with AD will be recruited and screened from 10 tertiary hospitals in China from October 2023 to October 2025 by publishing recruitment announcements in newspapers or on information platforms of the hospital official websites, display boards outside the clinics, and information counters. All patients are required to sign informed consent forms before the study begins.

In this study, patients will be included if they meet the inclusion criteria as follows: (1) diagnosis of AD according to Williams diagnostic criteria, (2) age 18-55 years for both males and females, (3) a target lesion with a diameter of 2-10 cm located on the limbs or the trunk, (4) EASI score<6, and (5) skin area<5% body surface area before treatment. The exclusion criteria are as follows: (1) pregnant or lactating women; (2) patients who have undergone AD system treatment within 3 months or local treatment or phototherapy within the past 2 weeks at the time of recruitment; (3) patients with serious systemic diseases or disorders of the heart, lungs, liver, or kidneys or mental illness; (4) patients who are allergic to calcium supplements; (5) patients with systemic disease or an active skin condition (eg, psoriasis) that affects the evaluation; (6) patients with scars, birthmarks, tattoos, freckles, etc; (7) patients with bacterial, viral, or fungal infections requiring anti-infective therapy; (8) patients who have participated or are currently participating in clinical trials of other drugs within the past month; and (9) patients with communication barriers, unable to complete the questionnaire.

### Randomization and Allocation Concealment

In this study, the block randomization method with a random combination of block lengths 4 and 6 will be used to assign the 200 patients with mild AD (1:1) to the treatment group (HBL with CAPCS; n=100, 50%) and the control group (HBL without CAPCS, placebo; n=100, 50%). Independent statisticians will use the entire randomization feature of SAS (SAS Institute) to construct 200 unique computer-generated random sequences for subsequent block randomization. The random numbers printed for participants will be placed in a light-resistant envelope and managed by a third party (not the investigators). If a patient is eligible after the baseline assessment and has provided informed consent, the clinical research coordinator will call the third party to obtain the patient’s random number and finish the group assignment. Next, a dermatologist will administer the corresponding intervention to the patient.

### Blinding

In this study, both patients with mild AD and dermatologists (investigators) will be blinded to the treatment allocation. Furthermore, the outcome assessors, data collectors, and statistician responsible for data analysis will also be unaware of the group assignment during the trial period to prevent bias.

### Intervention and Control

#### Treatment Group

Patients with mild AD in the treatment group will undergo 3 treatment sessions with HBL incorporated with CAPCS every day for 4 weeks. To ensure the correct application of HBL, we will provide individual guidance to each patient, instructing them that 1 knuckle length of HBL should be used to cover no more than 2% of the body surface area and to apply a 3-5-minute finger massage to promote its absorption. In this study, patients will also be instructed not to use any additional treatment for AD. If a patient uses additional treatment for an emergency, detailed information will be recorded and the patient will be asked to withdraw from the trial.

#### Control Group (Placebo)

**Figure 1:**
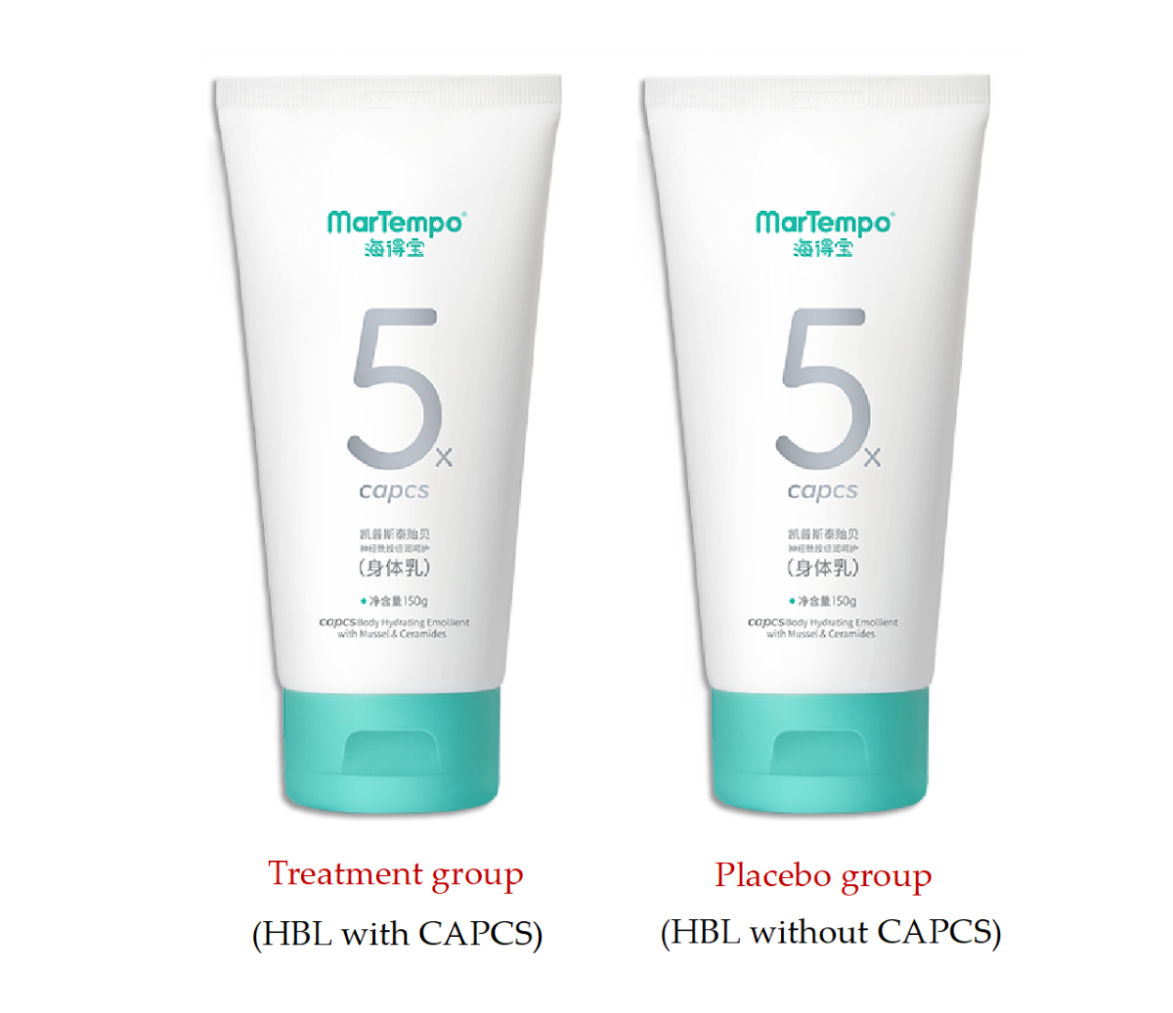
Images of HBL used for the 2 study groups. CAPCS: calcium-based antimicrobial peptide compounds; HBL: Haidebao Body Lotion.

Patients with mild AD in the control group will undergo 3 treatment sessions with the placebo (HBL without CAPCS) every day for 4 weeks. The HBL placebo is produced and provided by Shell Party Innovations Technology (Shenzhen) Co, Ltd, which maintains complete consistency in HBL in terms of appearance, weight, color, odor, and package, except for the incorporation of CAPCS (Figure 1). The application, individual guidance, and instruction regarding HBL placebo use in the control group will be completely in line with that in the treatment group. Patients in the control group will also be instructed not to use any additional treatment for AD. Like before, if a patient uses additional treatment for an emergency condition, detailed information will be recorded and the patient will be asked to withdraw from the trial.

#### Follow-Up

In this study, all patients will undergo a physical examination and skin lesion evaluation at baseline (day 0) following enrollment and then will be followed up for 4 weeks subsequently and undergo treatment efficacy evaluation at weeks 2 and 4. Detailed information about data collection, physical examination, and skin lesion evaluation is shown in Table 1.

**Table 1:** Detailed information about the timeline for data collection, physical examination, and skin lesion evaluation.

Study period	Enrollment (day 1)	Baseline (week 0)	Follow-up (week 2)	Follow-up (week 4)
Informed consent	Yes	N/A^a^	N/A	N/A
Screening	Yes	N/A	N/A	N/A
Clinical assessment	Yes	N/A	Yes	Yes
Medical history	Yes	N/A	Yes	Yes
Laboratory examination	Yes	N/A	N/A	Yes
Taking photos of lesions	N/A	Yes	Yes	Yes
NRS^b^ for itching	N/A	Yes	Yes	Yes
EASI^c^	N/A	Yes	Yes	Yes
DLQI^d^	N/A	Yes	Yes	Yes
Drug use	N/A	N/A	Yes	Yes
Adverse effects	N/A	N/A	Yes	Yes

^a^N/A: not applicable.

^b^NRS: Numeric Rating Scale.

^c^EASI: Eczema Area and Severity Index.

^d^DLQI: Dermatology Life Quality Index.

### Outcomes

#### Primary Outcome

In this study, the primary outcome is set as the proportion of patients who achieve EASI_60_ at week 2. The EASI_60_ is defined at least 60% improvement in the EASI score from baseline to week 2, which is calculated as (EASI at baseline – EASI at week t)/(EASI at baseline) × 100%.

#### Secondary Outcomes

##### EASI Score

The EASI score is commonly used to evaluate the condition of AD that is based on the severity of erythema (E), edema/papules/infiltration (I), desquamation (D), and lichenification (L) of the patient’s skin lesions and the affected area of involvement of the skin lesions in the head and neck (H), upper limbs (U), trunk (T), and lower limbs (L). The EASI score is calculated as H(E + I + D + L) × 0.1 + U(E + I + D + L) × 0.2 + T(E + I + D + L) × 0.3 + L(E + I + D + L) × 0.4, in which the affected area of involvement of the skin lesion (H, U, T, L) is evaluated through a 7-point Likert scale, with 0=0%, 1≤10%, 2=10%-29%, 3=30%-49%, 4=50%-69%, 5=70%-89%, and 6=90%-100%. The severity of erythema (E), edema/papules/infiltration (I), desquamation (D), and lichenification (L) is assessed through a 4-point Likert scale, with 0=none, 1=mild, 2=moderate, and 3=severe [27]. The EASI score ranges from 0 to 72, with a higher score suggesting that the patient is more ill. In this study, EASI scores will be assessed at baseline (week 0), week 2, and week 4 after treatment, and EASI50 and EASI75 scores will be assessed at weeks 2 and 4.

##### NRS Score

In this study, we will use the Numeric Rating Scale (NRS, score 0-10) to evaluate the degree of pruritus in patients with mild AD. The NRS is an 11-point Likert scale scored from 0=no pruritus to 10=extreme pruritus. The degree of pruritus will be categorized as no pruritus (NRS=0), mild pruritus (NRS=1-3), moderate pruritus (NRS=4-6), severe pruritus (NRS=7-8), and extremely severe pruritus (NRS=9-10) based on the NRS score [28,29]. In this study, we will assess the degree of pruritus in patients with mild AD before HBL application and at 1, 5, 10, 15, and 30 minutes following HBL application at baseline. We will also assess the degree of pruritus based on the NRS score once at weeks 2 and 4 after treatment.

##### IGA Score

The investigator’s global assessment (IGA) score will be used to estimate the overall condition of patients with mild AD. The IGA is a 6-point Likert scale, with 0=no erythema or inflammatory signs of AD, 1=minimal erythema and barely perceptible edema/papules/infiltration, 2=mild erythema and edema/papules/infiltration, 3=moderate erythema and edema/papules/infiltration, 4=severe erythema and edema/papules/infiltration, and 5=extreme skin lesions accompanied with severe erythema and edema/papules/infiltration [30]. In this study, the IGA score will be estimated at baseline (week 0), week 2, and week 4.

##### DLQI Score

The Dermatology Life Quality Index (DLQI) focuses on various impacts of skin diseases on patients in the last week, which mainly covers 7 dimensions: physiological response, psychological feeling, family, interpersonal communication, occupational restrictions, social activities, and treatment response [31]. The DLQI includes 10 items, all scored through a 4-point rating system of 0=none, 1=mild, 2=severe, and 3=extremely severe (see Table 2). The DLQI score is obtained by summing up the scores of each item, which ranges from 0 to 30, with a higher score suggesting lower dermatology life quality [32]. In this study, the DLQI score will be estimated at baseline (week 0), week 2, and week 4.

**Table 2:** Questions and answer options in the DLQI^a^.

Question number	Question (over the last week)	Options
1	How itchy, sore, painful, or stinging has your skin been?	a. None b. Mild c. Severe d. Extremely severe
2	How embarrassed or self-conscious have you been because of your skin?	a. None b. Mild c. Severe d. Extremely severe
3	How much has skin disease affected your shopping and household chores?	a. None b. Mild c. Severe d. Extremely severe
4	How much has your skin influenced the clothes you wear?	a. None b. Mild c. Severe d. Extremely severe
5	How much has your skin affected any social or leisure activities?	a. None b. Mild c. Severe d. Extremely severe
6	How much has your skin made it difficult for you to play any sport?	a. None b. Mild c. Severe d. Extremely severe
7	How much has your skin condition interfered with your ability to work or study?	a. None b. Mild c. Severe d. Extremely severe
8	How much has your skin created problems with your partner or any of your close friends or relatives?	a. None b. Mild c. Severe d. Extremely severe
9	How much has your skin caused any sexual difficulties?	a. None b. Mild c. Severe d. Extremely severe
10	How difficult is it for you during the treatment process, such as time delays?	a. None b. Mild c. Severe d. Extremely severe

^a^DLQI: Dermatology Life Quality Index.

##### Adverse Events

In this study, patients with mild AD will be informed of the risks that may arise in the study before they sign the informed consent form. We have defined adverse events as unintended signs, symptoms, or diseases occurring after treatment that are not necessarily related to the intervention. The occurrence of any adverse events will be accurately recorded in the case report form (CRF). In this study, appropriate treatment will be provided by dermatologists when they encounter adverse events, and the adherence condition of patients and adverse events will be recorded.

### Withdrawal and Dropout

According to the Declaration of Helsinki, all participants will be respected and can withdraw from the study at any time for any reason. The personal information about participants will be collected and kept confidential. In this study, patients can withdraw at any time, and the reason for withdrawal will be recorded in the CRF.

### Ethical Considerations

This study was reviewed and approved by the Ethics Committee of Shanghai Dermatology Hospital and the Institutional Ethics Review Committee of the Shanghai Skin Diseases Hospital in 2023 (approval number 2023-33). The implementation of the study will strictly adhere to the Declaration of Helsinki. Any adverse events, violations, and amendments to the study protocol or informed consent form will be reported to the Ethics Committee of the Shanghai Skin Diseases Hospital. All patients will sign the informed consent form before enrollment, and participant information will be protected.

### Quality Control

In this study, we will implement a series of measures to improve data quality and ensure the reliability of collected data. First, we will implement a pilot study to train all investigators participating in this study and to evaluate the reliability and validity of the CRF used for data collection. Second, we will assign two PhD students as the inspectors to conduct comprehensive checks in order to ensure the accuracy and integrity of the collected data. Third, we will actively contact all recruited patients during the 4 weeks of follow-up because frequent communication will enhance the patients’ trust and thereby increase their willingness to participate and remain in the study.

### Statistical Analysis

In this study, all data will be recorded in the CRF and then input into a database established using Epidata 3.1 software. The full analysis set (FAS) and the per protocol set (PPS) will be analyzed using the SAS 9.2 statistical package, and missing data will be processed using the multiple imputation method. In this study, quantitative variables with a normal distribution will be expressed as means (SDs), while quantitative variables with a skewed distribution will be expressed as medians (IQRs). Qualitative variables will be expressed as frequency counts and proportions (%). We will apply the 2-sample Student *t* test or the Wilcoxon rank-sum test to examine differences between groups for quantitative variables, as appropriate, and repeated measurement data will be analyzed using repeated-measure ANOVA. The Wilcoxon rank-sum test, the chi-square test, or the Fisher exact test will be performed to examine the differences between groups for qualitative variables, as appropriate. In this study, *P*<.05 (2-tailed) will be considered statistically significant.

## Results

**Figure 2:**
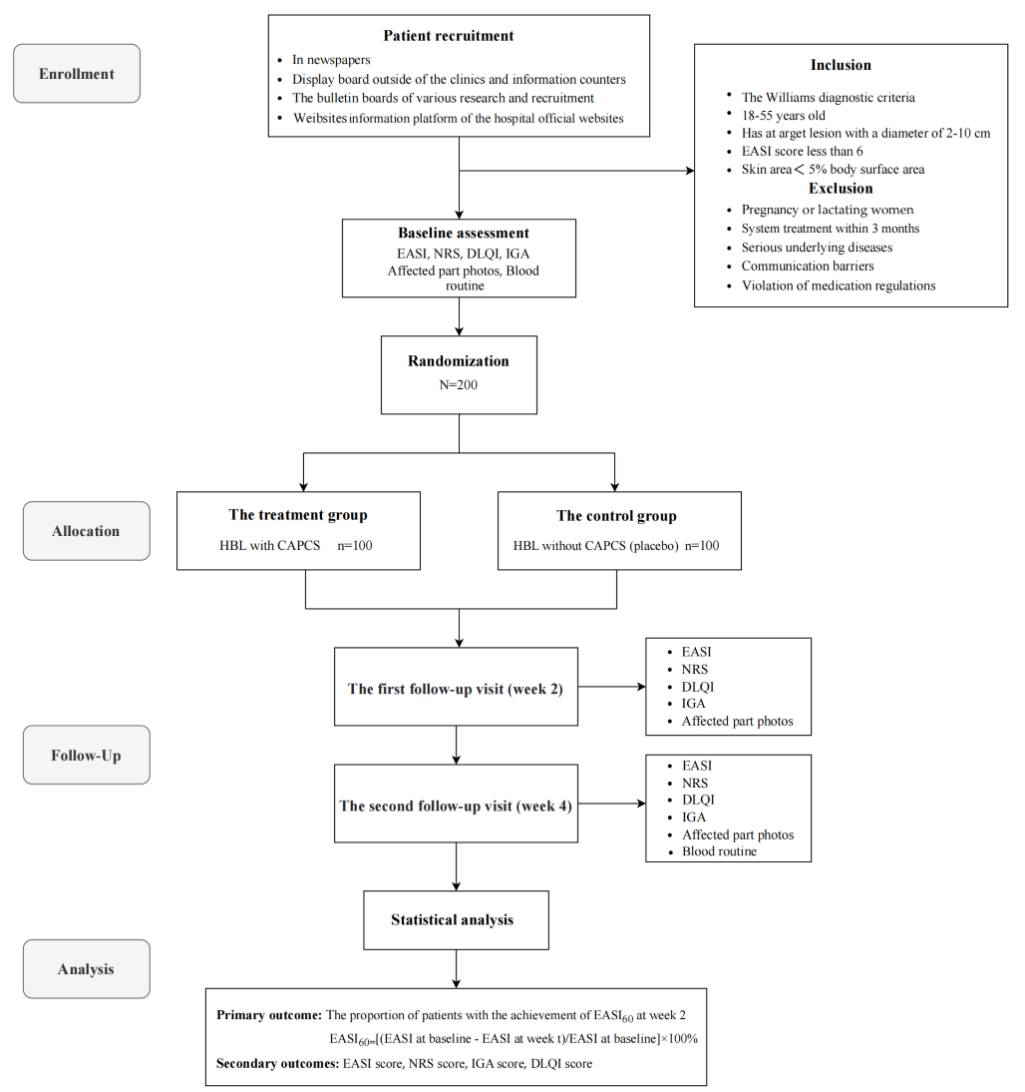
Study flowchart. CAPCS: calcium-based antimicrobial peptide compounds; DLQI: Dermatology Life Quality Index; EASI: Eczema Area and Severity Index; HBL: Haidebao Body Lotion; IGA: investigator’s global assessment; NRS: Numeric Rating Scale.

The flowchart of this study is shown in Figure 2. Participant recruitment began in January 2024 and is proposed to be finished by July 2025. As of May 2025, we enrolled 180 patients, with 160 (88.9%) completing the 2-week follow-up. Data collection and management are still ongoing, and data analysis has not yet been performed.

## Discussion

### Summary

AD is an autoimmune inflammatory skin disease that can last for a lifetime [33]. AD usually exhibits a chronic course, with thickening of the skin, sleep disturbance, severe life quality interference, and daily activity deprivation, which not only causes physical pain for the patient but also imposes heavy social and economic burdens on them [34,35]. For AD treatment, anti-inflammatory agents, wet-wrap therapy, systemic immunosuppressants, and short-term steroid treatment are used to manage persistent or severe AD cases [11]. However, the use of local and systematic treatment often fails to provide long-term disease control due to safety concerns. As adjuvant therapy, the external application of moisturizing emollients is the basis for AD treatment, which can not only improve the hydration of the cuticle and reduce inflammation [15] but also repair the damaged skin barrier, decrease the stimulation of external factors, and reduce the attack frequency and disease severity [16]. In recent years, the development of new active ingredients has led to the widespread clinical application of emollients to improve the skin barrier in patients with AD.

CAPCS, as new active ingredients, are derived from farmed marine shellfish. Previous studies indicate that the active calcium in CAPCS can inhibit the release of inflammatory mediators and exhibit anti-inflammatory and anti-allergic properties. Moreover, HBL incorporated with CAPCS has similar therapeutic effects as desonide cream in scratch control and itching alleviation in mice and in patients with AD [21], but there is a lack of high-quality clinical trial evidence. This protocol outlines the basic principle and design of a multicenter, double blind, randomized, placebo-controlled trial with 200 patients with mild AD in China. We plan to evaluate the treatment efficacy and safety of HBL incorporated with CAPCS in the enrolled patients, which is expected to yield more generalizable evidence due to the diversity in geography, climate, and participants from the 10 selected hospitals. To the best of our knowledge, this is the first multicenter clinical trial that will recruit 200 patients with AD to evaluate the treatment efficacy and safety of HBL incorporated with CAPCS in China. The clinical data of patients with AD will be extracted from the health information system (HIS) directly without recall bias, resulting in high data quality.

### Limitations

This study may have a few limitations. First, the study period is set to 4 weeks, which limits the observation of the long-term effects of HBL incorporated with CAPCS, as well as its efficacy in prevention of disease recurrence in patients with AD. Second, differences in climate (temperature, humidity, ultraviolet radiation, seasonal changes), lifestyle habits (high-fat food consumption, high-calorie diets, tobacco smoking, alcohol drinking) and other extrinsic factors (cultural differences, occupational exposure, etc) in the 10 selected hospitals may cause information bias, which should be adjusted through sensitivity analysis and subgroup analysis. Third, patients in this study will be instructed not to use any additional treatment for AD, which might induce a relatively high rate of withdrawal in them.

### Conclusion

This study is expected to confirm that the application of HBL incorporated with CAPCS can relieve itching, control scratching, and improve the skin barrier with reparative, anti-inflammatory, and moisturizing effects. If the treatment efficacy is proven, HBL incorporated with CAPCS could be clinically used as an adjunctive therapy in ameliorating mild AD.

## Abbreviations

AD: atopic dermatitis
AMP: antibacterial peptide
CAPCS: calcium-based antimicrobial peptide compounds
CRF: case report form
DLQI: Dermatology Life Quality Index
EASI: Eczema Area and Severity Index
FAS: full analysis set
HBL: Haidebao Body Lotion
IGA: investigator’s global assessment
NRS: Numeric Rating Scale
PPS: per protocol set

## References

[1] Gatmaitan JG, Lee JH (2023). Challenges and future trends in atopic dermatitis. Int J Mol Sci.

[2] Simon D, Wollenberg A, Renz H, Simon H (2019). Atopic dermatitis: Collegium Internationale Allergologicum (CIA) update 2019. Int Arch Allergy Immunol.

[3] Kapur S, Watson W, Carr S (2018). Atopic dermatitis. Allergy Asthma Clin Immunol.

[4] Siegfried E, Hebert A (2015). Diagnosis of atopic dermatitis: mimics, overlaps, and complications. J Clin Med.

[5] Silverberg JI, Gelfand JM, Margolis DJ, Boguniewicz M, Fonacier L, Grayson MH, Simpson EL, Ong PY, Chiesa Fuxench ZC (2018). Patient burden and quality of life in atopic dermatitis in US adults: a population-based cross-sectional study. Ann Allergy Asthma Immunol.

[6] Huang I, Chung W, Wu P, Chen C (2022). JAK-STAT signaling pathway in the pathogenesis of atopic dermatitis: an updated review. Front Immunol.

[7] Weidinger S, Beck LA, Bieber T, Kabashima K, Irvine AD (2018). Atopic dermatitis. Nat Rev Dis Primers.

[8] Coutanceau C, Stalder J (2014). Analysis of correlations between patient-oriented SCORAD (PO-SCORAD) and other assessment scores of atopic dermatitis severity and quality of life. Dermatology.

[9] Bekić S, Martinek V, Talapko J, Majnarić L, Vasilj Mihaljević M, Škrlec I (2020). Atopic dermatitis and comorbidity. Healthcare (Basel).

[10] Malik K, Heitmiller KD, Czarnowicki T (2017). An update on the pathophysiology of atopic dermatitis. Dermatol Clin.

[11] Eichenfield LF, Ahluwalia J, Waldman A, Borok J, Udkoff J, Boguniewicz M (2017). Current guidelines for the evaluation and management of atopic dermatitis: a comparison of the Joint Task Force Practice Parameter and American Academy of Dermatology guidelines. J Allergy Clin Immunol.

[12] Jang YH, Choi E, Lee H, Woo J, Park S, Noh Y, Jeon J, Yoo E, Shin J, Lee YW (2024). Long-term use of oral corticosteroids and safety outcomes for patients with atopic dermatitis. JAMA Netw Open.

[13] Frazier W, Bhardwaj N (2020). Atopic dermatitis: diagnosis and treatment. Am Fam Physician.

[14] Schmitt J, Buske-Kirschbaum A, Tesch F, Trikojat K, Stephan V, Abraham S, Bauer A, Nemat K, Plessow F, Roessner V (2018). Increased attention-deficit/hyperactivity symptoms in atopic dermatitis are associated with history of antihistamine use. Allergy.

[15] Denda M, Sato J, Tsuchiya T, Elias PM, Feingold KR (1998). Low humidity stimulates epidermal DNA synthesis and amplifies the hyperproliferative response to barrier disruption: implication for seasonal exacerbations of inflammatory dermatoses. J Invest Dermatol.

[16] Rajkumar J, Chandan N, Lio P, Shi V (2023). The skin barrier and moisturization: function, disruption, and mechanisms of repair. Skin Pharmacol Physiol.

[17] Catherine Mack Correa M, Nebus J (2012). Management of patients with atopic dermatitis: the role of emollient therapy. Dermatol Res Pract.

[18] Araviiskaia E, Pincelli C, Sparavigna A, Luger T (2022). The role of a novel generation of emollients, 'Emollients Plus', in atopic dermatitis. Clin Cosmet Investig Dermatol.

[19] Zhu J, Wang J, Wang S (2023). A single-center, randomized, controlled study on the efficacy of niacinamide-containing body emollients combined with cleansing gel in the treatment of mild atopic dermatitis. Skin Res Technol.

[20] Lee SE, Lee SH (2018). Skin barrier and calcium. Ann Dermatol.

[21] Mollanazar NK, Smith PK, Yosipovitch G (2016). Mediators of chronic pruritus in atopic dermatitis: getting the itch out?. Clin Rev Allergy Immunol.

[22] Jiang QY, Chen L, Jiang L (2018). Preparation and property of a bio-composite material with antibacterial ability and adsorption. Chin J Biol.

[23] Haney E, Mansour S, Hancock R (2017). Antimicrobial peptides: an introduction. Methods Mol Biol.

[24] Liu Q, Li M, Wang N, He C, Jiang X, Li J (2022). Calcium-based antimicrobial peptide compounds attenuate DNFB-induced atopic dermatitis-like skin lesions via Th-Cells in BALB/c mice. Int J Mol Sci.

[25] Wang N, Li M, Li J, Jiang X (2021). Efficacy of a cooling gel containing calcium-based antimicrobial peptide compounds combined with desonide cream in the treatment of atopic dermatitis in children: a randomized, double-blind controlled clinical study. Chin J Dermatol.

[26] Williams HC, Burney PG, Strachan D, Hay RJ (1994). The U.K. Working Party's Diagnostic Criteria for Atopic Dermatitis. II. Observer variation of clinical diagnosis and signs of atopic dermatitis. Br J Dermatol.

[27] Hanifin JM, Baghoomian W, Grinich E, Leshem YA, Jacobson M, Simpson EL (2022). The Eczema Area and Severity Index—a practical guide. Dermatitis.

[28] Silverberg JI (2021). Validity and reliability of a novel numeric rating scale to measure skin-pain in adults with atopic dermatitis. Arch Dermatol Res.

[29] Rams A, Baldasaro J, Bunod L, Delbecque L, Strzok S, Meunier J, ElMaraghy H, Sun L, Pierce E (2024). Assessing itch severity: content validity and psychometric properties of a patient-reported pruritus numeric rating scale in atopic dermatitis. Adv Ther.

[30] Siegfried E, Korman N, Molina C, Kianifard F, Abrams K (2006). Safety and efficacy of early intervention with pimecrolimus cream 1% combined with corticosteroids for major flares in infants and children with atopic dermatitis. J Dermatolog Treat.

[31] Finlay AY, Khan GK (1994). Dermatology Life Quality Index (DLQI)--a simple practical measure for routine clinical use. Clin Exp Dermatol.

[32] Basra M, Fenech R, Gatt R, Salek M, Finlay A (2008). The Dermatology Life Quality Index 1994-2007: a comprehensive review of validation data and clinical results. Br J Dermatol.

[33] Eichenfield LF, Stripling S, Fung S, Cha A, O'Brien A, Schachner LA (2022). Recent developments and advances in atopic dermatitis: a focus on epidemiology, pathophysiology, and treatment in the pediatric setting. Paediatr Drugs.

[34] Talamonti M, Galluzzo M, Silvaggio D, Lombardo P, Tartaglia C, Bianchi L (2021). Quality of life and psychological impact in patients with atopic dermatitis. J Clin Med.

[35] Cork MJ, Danby SG, Ogg GS (2020). Atopic dermatitis epidemiology and unmet need in the United Kingdom. J Dermatolog Treat.

